# Investigating the threat to Sitka spruce from *Ips typographus* : discrimination and colonization of Britain's principal commercial conifer by a damaging forest pest

**DOI:** 10.1002/ps.8644

**Published:** 2025-01-27

**Authors:** Daegan Inward, Jozsef Vuts, Gareth Thomas, Kerry Barnard, John C. Caulfield, Stephen J. Powers, Ana Uglow, Katy Reed

**Affiliations:** ^1^ Forest Research Alice Holt Lodge Farnham UK; ^2^ Protecting Crops and the Environment Rothamsted Research Harpenden UK; ^3^ Stats Powers Ltd Somerset UK

**Keywords:** bark beetle, invasive species, host choice, behavioral olfactometry, antennal electrophysiology, volatile organic compounds

## Abstract

**BACKGROUND:**

*Ips typographus* (L.), the eight‐toothed spruce bark beetle (Coleoptera: Scolytinae), has devastated European Norway spruce (*Picea abies*) forests in recent years. For the first time, *I. typographus* has established localized breeding populations in Britain, where Sitka spruce (*P. sitchensis*) is a critical component of plantation forestry. The interactions between Norway spruce and *I. typographus* are well understood, but relatively little is known about the susceptibility of Sitka spruce to the beetle. This study aimed to determine whether *I. typographus* would select Sitka, compared to Norway spruce, as a host for breeding, and to study the chemical ecology underlying these host preferences.

**RESULTS:**

Host choice assays were conducted in the laboratory using freshly cut spruce logs, and then verified in the field in an area with an endemic population of *I. typographus*. Overall, colonization and breeding success were found to be similar in cut Sitka and Norway spruce material. The response of *I. typographus,* reared on both Norway and Sitka spruce, to headspace extracts of aged and fresh Norway and Sitka spruce material was tested behaviorally using four‐arm olfactometry. Odors of aged wood from the two species were equally attractive, and fresh Sitka was more attractive than fresh Norway spruce. Antennal responses to Norway Spruce and Sitka Spruce headspace extracts were located using GC‐EAG and identified by coupled GC‐mass spectrometry and GC co‐injection with authentic standards. Norway‐ and Sitka spruce‐reared beetles did not differentiate between synthetic Norway or Sitka spruce blends and responded similarly.

**CONCLUSION:**

These findings suggest *I. typographus* will select and colonize cut Sitka as readily as cut Norway spruce, with implications for its establishment risk in Sitka‐growing regions. Whilst the susceptibility of live Sitka trees remains unclear, the study advances the understanding of the role of both host‐emitted volatile organic compounds (VOCs) in primary host location and induced host preference in host selection by *I. typographus.* © 2025 Crown copyright and The Author(s). *Pest Management Science* published by John Wiley & Sons Ltd on behalf of Society of Chemical Industry. This article is published with the permission of the Controller of HMSO and the King's Printer for Scotland.

## Graphical abstract

The forest pest *Ips typographus* was found to demonstrate attraction to Sitka spruce, and to colonize and breed upon cut logs as successfully as its primary host Norway spruce. This has implications for the beetle's establishment and impact in Sitka‐growing regions, including Britain where *I. typographus* has recently established for the first time.

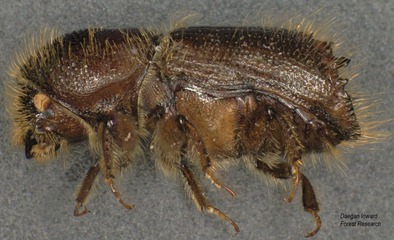

## 1 INTRODUCTION

The eight‐toothed spruce bark beetle, *Ips typographus* (L.), (Coleoptera: Scolytinae), is the most destructive forest pest in Europe, and is widely present throughout the natural and planted range of spruce trees across the Palearctic region. Unprecedented populations of *I. typographus* have developed in Norway spruce (NS) forests *Picea abies* L. (Pinaceae) across continental Europe over recent years, driven by successive years of high temperatures and drought, which have weakened the trees and made them susceptible to mass attack by the beetle.^1^ Over 100 million m^3^ of NS have been killed by bark beetle attack across Europe since 2013.^2^ Following the first detection of *I. typographus* in Kent, England, in 2018, at least 44 isolated incursions (localized breeding populations) of this non‐native beetle have been subsequently detected on windthrown NS trees in southeast England,^3^ and recently in East Anglia for the first time.^4^ Most incursions are thought to have been initiated in early June 2021, when the very large population of *I. typographus* present in northern France and Belgium apparently undertook a synchronized dispersal flight and suitable weather conditions assisted beetles to penetrate more than 160 km into England.^5^ Despite being frequently intercepted with imported spruce wood,^6^ *I. typographus* had not previously established in Britain.

As a regulated quarantine pest in the UK, eradication measures have been applied to all of the detected incursions of *I. typographus*, and a large, demarcated area was created (and subsequently expanded) in southeastern England to limit movement of susceptible *Picea* material.^4^ Repeated detections of the pest in pheromone traps since 2019,^7^ and evidence that the beetle is capable of naturally dispersing across the English Channel,^5^ suggest, however, that further establishments of the pest *via* the same pathway may be expected. Consequently, a transition away from growing spruce in southern Britain is being implemented, including a prohibition on the planting of spruce trees within the demarcated zone.^4^ In the north and west of Britain, however, the forest industry is notably reliant upon the growing of Sitka spruce (SS) (*P. sitchensis* Bong.; Carr.), which comprises around 54% of all planted conifers by area, with over 500 000 ha planted in Scotland alone.^8^ The native range of SS comprises the Pacific west coast of USA and Canada, so the species grows vigorously in the maritime climate of Britain and Ireland. Its productivity and resilience to damage from pests and disease have made SS the most economically important conifer species planted in the British Isles, additionally sequestering large amounts of carbon each year.^9^

Although the impacts of *I. typographus* upon NS are well understood, little is known about the suitability or attractiveness of SS as a host for the beetle, or how resistant it may be to attack. Although SS has been planted in France, Germany, Denmark and Norway (on a considerably smaller scale than in the British Isles^10^), no outbreaks of the beetle in these plantations have been reported to date. SS trees evidently killed by *I. typographus* have been identified in Denmark, although uncertain predisposing factors such as butt‐rot fungi may have assisted in the trees' mortality (Inward D, pers. obs.). Breeding activity by *I. typographus* was also recently detected on two felled Sitka spruce trees in southern England (Inward D, pers. obs.,^4^) in close proximity to a larger number of infested Norway spruce trees assumed to be the source of the colonizing individuals. Flø *et al*.^11^ compared the reproductive performance of *I. typographus* upon NS, SS, and the hybrid Lutz spruce (*Picea glauca* × *P. sitchensis*), by caging adults onto cut logs and assessing breeding success. More breeding galleries were created in NS logs than the novel hosts, but the number and size of offspring per gallery were similar across spruce types. This suggests that the nutritional quality of the hosts was comparable, but acceptance or establishment by the adults was reduced in SS and Lutz spruce. Such ‘no‐choice’ techniques may artificially influence adult acceptance of a novel species, and further investigations into *I. typographus* selection and utilization of SS are needed.

*Ips typographus* uses volatile organic compounds (VOCs) to identify host and non‐host trees,^12^, ^13^ which can be located through primary and secondary attraction. Primary attraction is the initial phase, in which beetles locate host trees using visual and olfactory stimuli over long‐ and short‐distances.^14^ Secondary attraction involves male and female beetle aggregation in response to pheromone production by beetles that have already colonized the tree.^15^ Beetle populations can be present at endemic levels, where low numbers specifically target stressed or weakened trees, or epidemic levels, where numbers increase and adjacent, healthy trees may be successfully colonized.^14^ Previous work shows that NS VOCs, as well as non‐host VOCs, are detected by the antennae of *I. typographus*,^16^ suggesting a potential role for VOCs in host location. Five oxygenated NS monoterpenes were shown to elicit antennal responses in both male and female *I. typographus*.^17^ One compound, trans‐4‐thujanol, demonstrated dose‐dependent repellence against the beetle, indicating a potential role in anti‐herbivore defence,^18^ and is an anti‐attractant for females to a potency comparable to other known anti‐attractants.^19^ Interestingly, *I. typographus* appear to demonstrate a preference for drought‐stressed NS bark, indicating that beetles prefer trees in a physiologically weakened state.^1^ Several studies indicate *I. typographus* can detect non‐host VOCs, in addition to host VOCs, which may act as long‐range discriminatory cues to differentiate between host and non‐host species.^20^, ^21^, ^22^ Whilst the role of NS VOCs in host recognition by *I. typographus* has been suggested, the differences in VOC production between NS and SS, and beetle behavior towards SS, remains an important knowledge gap.

There is a clear risk of *I. typographus* permanently establishing in Britain and spreading to SS‐growing regions. As observed across Europe, climate change may promote population growth of the beetle through reduced generation time, as well as altering the health and resistance of their host trees, but the threat to SS remains unclear. The principle aim of this study was therefore to establish whether *I. typographus* would select SS trees, compared to NS trees, as suitable hosts for breeding, and to understand the semiochemistry underlying host selection mechanisms. Laboratory and field‐based choice experiments compared the beetles' host selection preferences between NS and SS cut material. To provide a mechanistic understanding of the response of the beetles to VOCs emitted by SS as compared to NS, behavioral olfactometry and antennal electrophysiology assays, simulating the ‘host search’ phase of host selection, were additionally conducted. Sitka spruce has proved a highly productive forestry tree in Europe, little affected by pests and diseases to date. Improved understanding of the potential interactions with *I. typographus* now seems critical for assessment of the future risks and value of Sitka spruce in European forestry.

## 2 MATERIALS AND METHODS

### 2.1 Plant material

Norway spruce (NS) and Sitka Spruce (SS) trees were sourced from a mixed species trial plot in Alice Holt forest, Surrey (51.176897° N, −0.846594° W), with additional SS material sourced from Bellever forest, Dartmoor, Devon (50.588958° N, −3.914438° W), where it is planted under more typical damp, cool conditions. Billets (small log sections, 10–14.5 cm diameter, 27 cm long) for choice experiments were cut from freshly felled NS and SS trees (~ 20 cm diameter at breast height (DBH)) as required throughout the year. Cut billets were stored in an unheated outbuilding to protect them from colonization by other insects for 14–57 days. For chemical ecology studies, the olfactometry and volatile collection work was conducted with billet material collected in summer 2020 and 2021. Due to operator availability, billets for volatile samples for GC‐EAD were collected from four freshly felled NS from Surrey and four SS trees from Dartmoor, UK, in December 2021. Material from each tree was then either assayed in olfactometer tests immediately (fresh) or retained for 2 weeks before assaying (aged).

### 2.2 Insects

A breeding colony of *Ips typographus* was maintained and cycled upon fresh‐cut NS logs in large clip‐top sealed boxes ventilated with drilled (~1 mm) holes. Beetles were additionally reared on SS logs for chemical ecology studies. Rearing and choice experiments were carried out under quarantine laboratory conditions at Alice Holt research station, Surrey, as required for a UK‐regulated quarantine pest, in a constant temperature (CT) room at 20–22  °C with a 16:8 light: dark cycle. For chemical ecology studies, newly emerged adult beetles were sourced from the breeding colony; behavioral and electrophysiological experiments were carried out with both NS‐ and SS‐reared beetles because of potential induced host preference effects.^23^, ^24^, ^25^

### 2.3 Laboratory choice experiments

NS/SS choice experiments were set up in eight blocks from March to December 2019, at one or two timepoints and 5–15 replicates per block, according to the availability of newly emerged and dispersal‐ready adult beetles. Experimental blocks 1–7 used billets from 1 to 3 trees with both NS and SS felled at Alice Holt. Block 8 paired NS trees from Alice Holt with four SS trees sourced from Dartmoor to investigate whether SS growing conditions influenced beetle selection of host material. The choice experiments were conducted in the CT room within 13 L food storage containers (31 × 31 × 16 cm, Stewart sealfresh), ventilated with approximately 1 mm drilled holes and with a thin layer of sand at the bottom of the containers. One billet each of NS and SS, matched in diameter, was placed in a container. Between 6 and 12 beetles (according to availability from the reared colony) were placed between the billets and allowed to select and colonize either billet. We assumed a 1:1 sex ratio emerging from the colony,^26^ and added a mixed group of males and females in each case to simulate colonization choices being made by a natural field population. Any dead beetles observed in the containers after 4 days were removed and replaced with live ones. To assess colonization by the beetles, after 4–5 weeks the bark was cut lengthwise using a Stanley knife and carefully peeled from the billet using a curved bark peeler (Morris of Dunford). The inner surface of the peeled bark was digitally photographed and the number of adult galleries with and without egg notches and/or larval galleries (defined as ‘breeding galleries’ and ‘non‐breeding galleries’, respectively) was counted at the time of assessment and verified in ImageJ using the digital photograph.

### 2.4 Field choice experiment

**Fig. 1:**
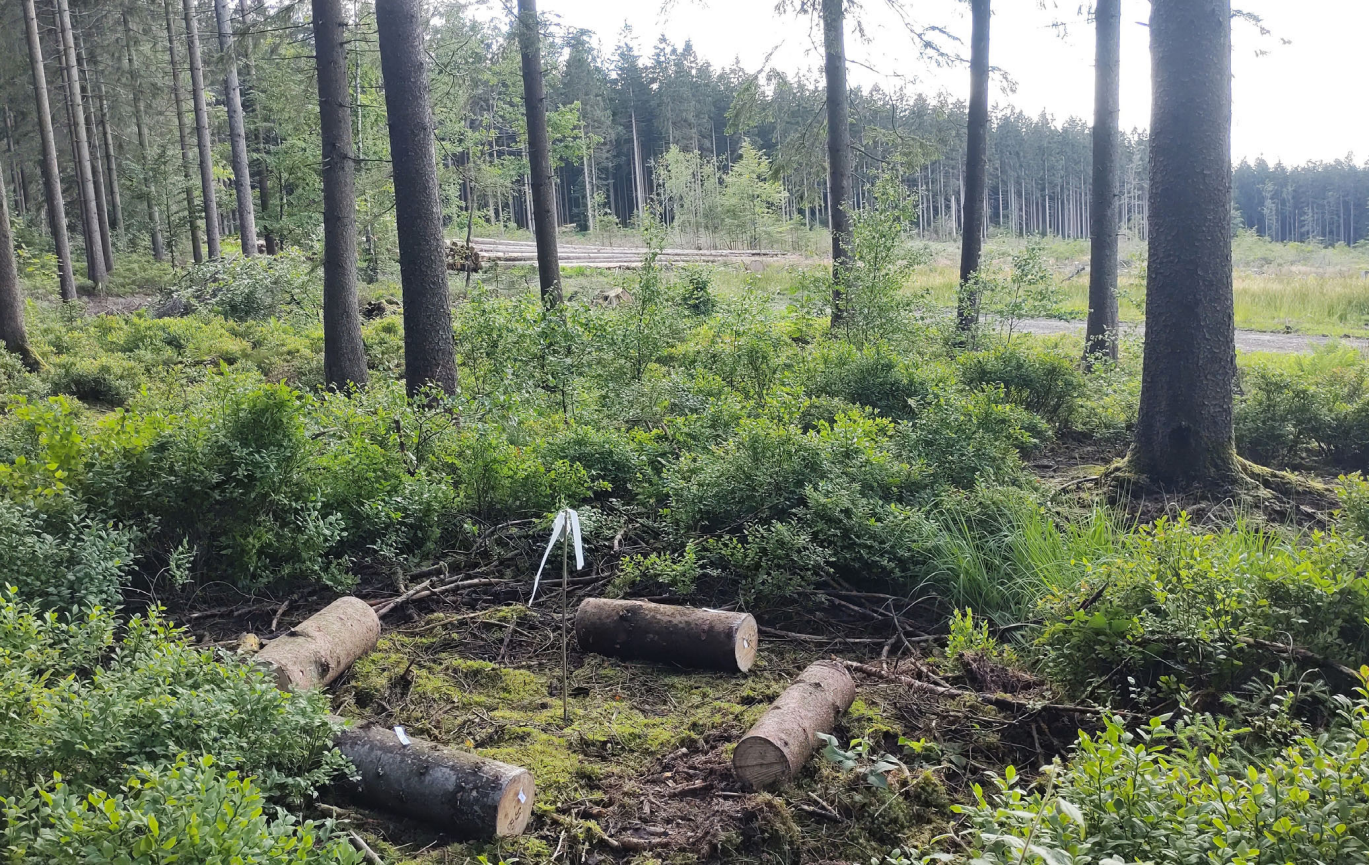
A plot from the field choice experiment. Fresh‐cut Norway spruce and Sitka spruce logs are arranged in an alternating ‘open’ square, with a low‐dose pheromone lure attached to a central post. This plot is located at the forest edge; the timber stack is visible in the background in corner of the clearfell.

Four NS trees from Alice Holt and four SS trees from Dartmoor (~ 20–25 cm DBH) were felled in mid‐June 2022 and cut into 60 cm long logs (8–22 cm diameter). The ends were sealed with paraffin wax and the logs stored outdoors in the shade to reduce desiccation and screened with fine nylon mesh to prevent colonization by insects. Bark moisture content was calculated from the fresh and oven‐dried weight of bark samples removed with a 15 mm arch punch. After 10 days, the logs were transported to a NS forest site near Willerzie in the Belgian Ardenne (49.957367° N, 4.832081° E), an area which had been subject to sustained attack by *I. typographus* over several years (Tavier G, Service public de Wallonie, pers. comm.). The site had been clear‐felled the previous year and was bordered by mature planted NS forest on the south and west sides. The experiment was timed to coincide with the emergence of new adults emerging from a NS timber stack located at the southwest corner of the clearfell. Nine pairs of level, open plots were selected at regular intervals of 20–25 m along the forest/clearfell edge (18 total, each ~ 2.5 m^2^). Each plot pair consisted of one in the clearfell, and the other about 5 m into the forest edge (shaded from the sun), forming two parallel L‐shaped transects with the log stack at the center. Distance from the timber stack to the plots ranged from 31 to 133 m. Within each plot, 2 × NS and 2 × SS logs were laid out to form an alternating square, at the center of which a low‐dose pheromone lure was attached to a post at 50 cm above ground level (Fig. 1). Lures consisted of a cellulose wick impregnated with 40 mg of (*S*)‐*cis*‐verbenol (Merck, 95% purity) diluted in 1 mL of 2‐methyl‐3‐buten‐2‐ol (Merck, 98% purity), mimicking the *I. typographus* aggregation pheromone.^27^ This was contained within two sealed 50 μm‐thick polythene ziplock bags (6 × 4 and 8 × 6 cm). In an open‐air calibration experiment in early June, this arrangement provided a mean release rate of 11 mg/day, over 6 days. In alternate plots, the logs were laid out in a ‘closed’ square (billets 50 cm from the central lure), or ‘open’ square (each 1 m from the lure). Each square was oriented so that 1 NS and 1 SS log ‘pointed’ towards the timber stack and was cleared of large woody debris and undergrowth to create a uniform surface for arriving beetles to traverse and select a log. The logs were assessed for beetle colonization in late August 2022, by which time the next generation had mostly completed development due to very warm weather. External entry and emergence holes made by adult *I. typographus* were counted (identified by their size, being visibly larger than ventilation holes made along the maternal galleries), and the bark was carefully peeled and photographed as described above. The number and length of breeding galleries and number of dead / failed pupae present were recorded from the photographs using ImageJ. Between 5 and 20 new adults were collected from each log where present and stored in 70% IMS before being photographed in the laboratory using a GXCAM U3‐18 camera (GT Vision Ltd) fitted to a dissecting microscope. Beetle body length was measured using GXCapture‐T software v. 64 (GT Vision Ltd).

### 2.5 Chemical ecology investigations

#### 2.5.1 Dynamic headspace collection (air entrainment)

To establish which volatiles are responsible for the observed *I. typographus* behavioral choices, the headspace of aged (2 week‐old) and freshly cut (1 day‐old) NS and SS billets (using 41–61 g of material) was sampled by air entrainment (*n* = 4 for both spruce species and age classes). Charcoal‐purified air was pumped into the headspace of a glass chamber (23 cm h × 6.5 cm diam., Biochem Glass Apparatus Ltd., Milton Keynes, UK), attached to a metal plate with bulldog clips, at a rate of 600 mL/min, and subsequently pulled out at 500 mL/min through 50 mg Porapak Q 50/80 adsorbent polymer (Sigma‐Aldrich, Gillingham, UK), sandwiched between glass wool plugs in a glass tube (4 mm diam.). Each collection lasted for 24 h using established methods.^28^, ^29^ To note, synthetic blends prepared based on headspace extract compositions from corresponding behaviorally active billets evoked the same kind of response as the billets. This indicates that the behaviorally active compounds were captured by 24 h dynamic headspace collections in physiologically relevant ratios as were released by the billets, suggesting no volatile breakthrough occurred using the Porapak tubes. Trapped compounds were eluted from the adsorbent with 750 μL freshly distilled diethyl ether and the samples stored at −20  °C until analysis. See supplementary methods for more detailed information.

#### 2.5.2 Behavioral assays (olfactometry)

To gain a better understanding of the underlying mechanisms behind the field choice tests, we assumed that the beetles use volatile cues to locate spruce material, whereas contact chemical cues on the surface of the bark play a role in host acceptance. To investigate spruce log volatiles, a four‐arm olfactometer was used to measure *I. typographus* responses to spruce billet material odor and synthetic blends. The olfactometer consisted of three layers of Perspex, held together with plastic nuts and bolts. Both the top and bottom discs had a 156 mm diameter and 5 mm thickness, and the bottom disc was fitted with a filter paper base to provide traction for the walking insect. The middle part was 180 mm in diameter and 7 mm thick and was manufactured to embody four side areas or arms (55 mm in length × 5 mm height each) situated at 90° to each other. The side areas narrowed towards the perimeter and were connected *via* a 3 mm diameter hole at the end to either 1 L glass chambers with Teflon™ tubing or glass arms directly. Assuming that the billet is ‘attractive’, one of the glass chambers (50 cm^3^) contained a spruce billet and the other three chambers were empty and acted as controls. This setup ensured the robustness of the experiment by making it less likely for an insect to accidentally walk in or out of the treated region. In experiments testing synthetic blends, the side areas were connected to glass arms (narrow part: 50 mm length × 2.5 mm diameter, wide part: 90 mm length × 20 mm diameter). Before each experiment, all glassware was washed with Teepol detergent (Orpington, UK), rinsed with acetone and distilled water, and baked for 2 h at 160  °C. Perspex components were washed with Teepol solution, rinsed with 70% ethanol solution and distilled water and left to air‐dry. The olfactometer was illuminated from above by diffuse uniform lighting from two 18 W/35 white fluorescent light bulbs screened with red acetate. The device was surrounded by black paper to remove any external visual stimuli.

A single newly emerged beetle was introduced through a hole in the top of the olfactometer and was given 2 min to acclimatize (the room temperature was 20  °C and RH 60%), after which the experiment was run for 16 min. Analysis of sex ratios in three early olfactometer experiments indicates similar trends in behavioral response between females and males, which led us to decide not to separate the sexes (Supporting Information, Fig. S1). The air was drawn through the central hole by a vacuum pump and pulled through each of the four side arms (75 mL/min per arm) and subsequently exhausted from the room, which was the setup for using the glass side arms only. For the setup with holding chambers, air was pushed through Teflon tubing at 100 mL/min into each of the four chambers, which were connected to the four side areas of the olfactometer. The olfactometer was rotated by 90° every 2 min to control for any directional bias. The olfactometer was divided into five regions that corresponded to each of the four side arms and the central compartment, and the time spent in each area was recorded using specialist software (OLFA, Udine, Italy).

The following experiments were performed with both NS‐ and SS‐reared beetles: (i) aged (2 week‐old) NS billet *vs* controls (air), (ii) aged SS billet *vs* controls, (iii) aged NS *vs* SS billet *vs* controls, (iv) freshly cut (1 day‐old) NS *vs* SS billet *vs* controls, (v) synthetic NS blend *vs* SS blend (10 μL each) *vs* controls (10 μL diethyl ether), which were applied onto pieces of filter paper and placed into the glass arms connected to the olfactometer. Synthetic blends comprising commercially available EAG‐active components of aged NS and SS billet headspace extracts, their composition represented constituent ratios based on GC analysis and 10 μL of each blend corresponded to the amount of volatiles released by 1 g billet material in 1 h. Applied amounts (ng) for the NS blend: (*RS*)‐α‐pinene 107.4, (*RS*)‐ β ‐pinene 240.1, myrcene 165.3, 3‐carene 48.5, (*RS*)‐limonene 409.3, γ‐terpinene 2.1, terpinolene 15.1, (*RS*)‐camphor 0.5, (+)‐longifolene 6.9. Applied amounts (ng) for the SS blend: (*RS*)‐α‐pinene 41.0, (*RS*)‐ β ‐pinene 53.3, myrcene 129.3, 3‐carene 35.1, (*RS*)‐limonene 694.5, γ‐terpinene 3.4, terpinolene 42.2, (*RS*)‐camphor 0.7, (+)‐longifolene 0.2. All chemicals were purchased from Sigma‐Aldrich, UK. (>90% purity), apart from (+)‐longifolene (99% purity), which was from Fluka, UK.

#### 2.5.3 Gas chromatography‐flame ionization detector (GC‐FID) analysis

Collected headspace extracts were analyzed on an Agilent 6890A gas chromatograph (GC) equipped with a cool on‐column injector, a flame ionization detector and a 50 m × 0.32 mm ID, 0.52 μm film thickness HP‐1 column (Agilent, Santa Clara, CA, USA). The oven temperature was maintained at 30  °C for 1 min, then programmed to increase at 5  °C/min to 150  °C and held for 0.1 min, then programmed to increase at 10  °C/min to 250  °C and held for 20 min. The carrier gas was hydrogen (3.1 mL/min flow rate). Quantification of compounds was achieved by the single‐point external standard method with a series of C7‐C22 alkanes, where the amount of an analyte was estimated using the peak area of the nearest alkane peak, the amount of which was known.

#### 2.5.4 Coupled GC‐electroantennography (GC‐EAG) analysis

Electrophysiological responses from the antennae of NS‐ and SS‐reared *I. typographus* beetles to aged NS and SS billet headspace extracts were recorded using coupled GC‐electrophysiology (GC‐EAG)^30^ (Supplementary methods). A beetle was immobilized by chilling on ice, and one of its antennae removed and mounted between two glass capillary electrodes filled with ringer solution (without glucose) (7.55 g/L sodium chloride, 0.64 g/L potassium chloride, 0.22 g/L calcium chloride, 1.73 g/L magnesium chloride, 0.86 g/L sodium bicarbonate, 0.61 g/L sodium orthophosphate). Antennal signals were passed through a UN‐06 high‐impedance amplifier (Ockenfels Syntech GmbH, Germany). Separation of the volatiles collected from billets was achieved on an Agilent 6890 N GC (Agilent Technologies), equipped with a cool on‐column injector and a flame ionization detector (FID), using a 50 m × 0.32 mm ID, 0.52 μm film thickness HP‐1 column. The oven temperature was maintained at 30  °C for 2 min and then programmed to increase at 5  °C/min to 250  °C. The carrier gas was helium. The outputs from the EAG amplifier and the FID were monitored simultaneously and analyzed using a customized software package (Syntech GC/EAD for Windows v 2.3 09/1997). One μL aliquots of NS and SS volatile samples were analyzed. A compound was identified as EAG‐active if it evoked an antennal response in at least three of five coupled runs/spruce species/beetle host line.

#### 2.5.5 Coupled GC‐mass spectrometry (GC–MS) analysis

GC–MS analysis of collected headspace VOCs was performed using a Waters Autospec Ultima mass spectrometer, coupled to an Agilent 6890 GC fitted with a HP‐1 capillary column (50 m × 0.32 mm ID, 0.52 μm film thickness). Ionization was by electron impact (70 eV, source temperature 220 °C). Helium was the carrier gas. The oven temperature was maintained at 30 °C for 5 min and programmed to increase at 5 °C/min to 250 °C. Tentative identifications of EAG‐active peaks were made by comparison of mass spectra within the NIST 2008 mass spectral database^31^ and Kováts retention index (KI) values, as well as by GC peak enhancement *via* co‐injection with authentic standards.^32^

### 2.6 Statistical analyses

For lab choice experiments, statistical analyses to test the difference in colonization of the NS and SS billets and subsequent breeding gallery construction were carried out by fitting general linearized mixed‐effects models in R 4.2.1.^33^ The binomial error family was specified in models to compare the proportions on NS *vs* SS in each box of galleries (all), breeding galleries, and non‐breeding galleries. 0 and 1 were specified as explanatory variables in two models, model 0 and model 1. Both models included random effects for experimental setup timepoint, nested within setup block, NS tree, and SS tree. To compare breeding gallery lengths, responses were the total length of breeding galleries within each billet, where breeding galleries were present, and length of individual breeding galleries, square root‐transformed. Explanatory variables were species (NS *vs* SS), billet diameter and their interaction. Random effects were fitted as above, with further nested levels for replication and individual billet.

For the field choice experiment, the responses were totals per log, as follows: combined entry and emergence holes; number of breeding galleries; breeding gallery length; number of dead/failed pupae; and length of newly developed adults. The explanatory variables were species (NS or SS), moisture content, and log surface area (cm^2^), and their interactions; area type (clear‐felled or forest edge); block size (open or closed); and distance from the timber stack (m). Continuous explanatory variables were scaled and centered using the scale () function. Random effects were specified for block, nested within area type, and tree. Generalized linear mixed‐effects models for the negative binomial family were fitted in the GLMM package^34^ for all count data, and linear mixed‐effects models were fitted in the lme4 package^35^ for the gallery length data. Adult length data were fitted with linear mixed‐effects models with additional nested random effects specified for log within tree, and log within block within area type. Differences between the binomial models were tested using the ‘ANOVA’ function. Significance of the explanatory variables in all other models was checked using the ANOVA function in the car package.^36^ For each response, non‐significant explanatory variables were progressively removed to produce a minimal adequate model, with higher‐order interactions removed first. The initial and final models were checked for overdispersion, zero inflation and goodness‐of‐fit using the simulation output function in the DHARMa package,^37^ or by visually examining model residual plots. Post‐hoc testing was carried out in the emmeans package.^38^

For the olfactometry experiments, to account for the replication and areas within each replication as variance components in a split‐plot design, the method of residual maximum likelihood (REML) was used to fit a linear mixed model to the time spent data, nesting the areas within each replication and testing the treatment effect using an approximate *F*‐test. The data were analyzed after square root‐scale transformation to account for some heterogeneity of variance over the treatments. Means are presented with standard error of the difference (SED) values for their comparison, and the least significant difference (LSD) at the 5% (*P* = 0.05) level of significance was used for separation of means. Genstat^39^ was used for the analysis.

Differences in VOC composition (ng/g fresh weight/h) for the different treatments were analyzed, using analysis of variance (ANOVA), to determine the statistical significance of the effect of species, age and the interaction between these two factors. The analysis took account of the two samples from each tree, using a samples‐from‐main‐plots design for the experiment, thus testing the main effect of species at the level of individual trees, and the main effect of aging and the species by aging interaction at the level of samples within trees (Supplementary methods). Canonical variates analysis (CVA) was used to analyze all the chemical data together (on the log‐scale, as required from the ANOVA) and discriminate between the four treatment combinations (two species by two ages). The method finds linear combinations (i.e. canonical variates, CVs) of the chemicals that maximize the ratio of the between treatment combinations variation to the within treatment combinations variation, thus performing a discrimination between all treatment combinations. The fewest number of CVs are retained that take up the most variation in the data and hence make the most discrimination.

## 3 RESULTS

### 3.1 Lab choice experiment

**Figure 2:**
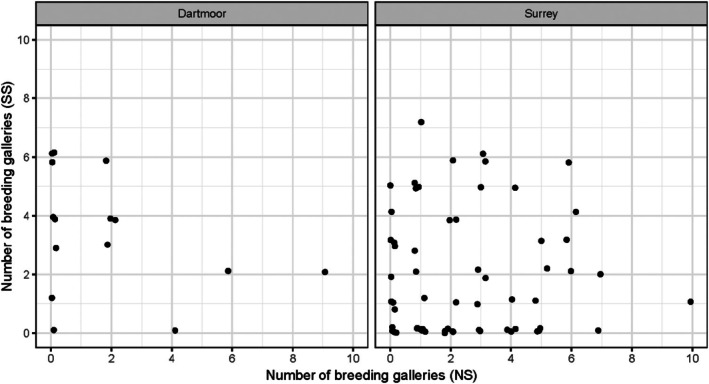
The number of breeding galleries per billet on Norway Spruce (NS) and Sitka Spruce (SS) in the laboratory host choice experiment, according to the source of the Sitka spruce billets (Dartmoor (*n* = 24 replicates) and Surrey (*n* = 103 replicates)).

Overall, colonization and breeding success by *I. typographus* were similar on billets of both spruce species, regardless of the location where the billets were sourced (Fig. 2). In total (from all treatments), 75/127 of the Norway spruce billets and 65/127 of the Sitka spruce billets exhibited feeding and/or breeding galleries. For 36/127 replicates (28%), breeding galleries were made in both NS and SS billets, and for 23 (18%), breeding galleries were made in neither; for 29 replicates (23%), breeding galleries were made in SS only, and for 39 replicates (31%), breeding galleries were made in NS only. In the analyses, host species (NS or SS) did not influence the proportion of breeding or non‐breeding galleries created in the billet pairs (Supporting Information, Table S1). While none of the explanatory variables influenced total (cumulative) breeding gallery length per colonized billet, the best fit models for individual breeding gallery lengths included host species, suggesting individual breeding galleries were longer in NS than SS (Supporting Information, Table S2). The difference between the average model‐predicted individual breeding gallery lengths (56.3 ± 4.5 mm and 48.0 ± 4.5 mm in NS and SS, respectively) was not significant in post‐hoc testing (Supporting Information, Table S2).

### 3.2 Field choice experiment

**Figure 3:**
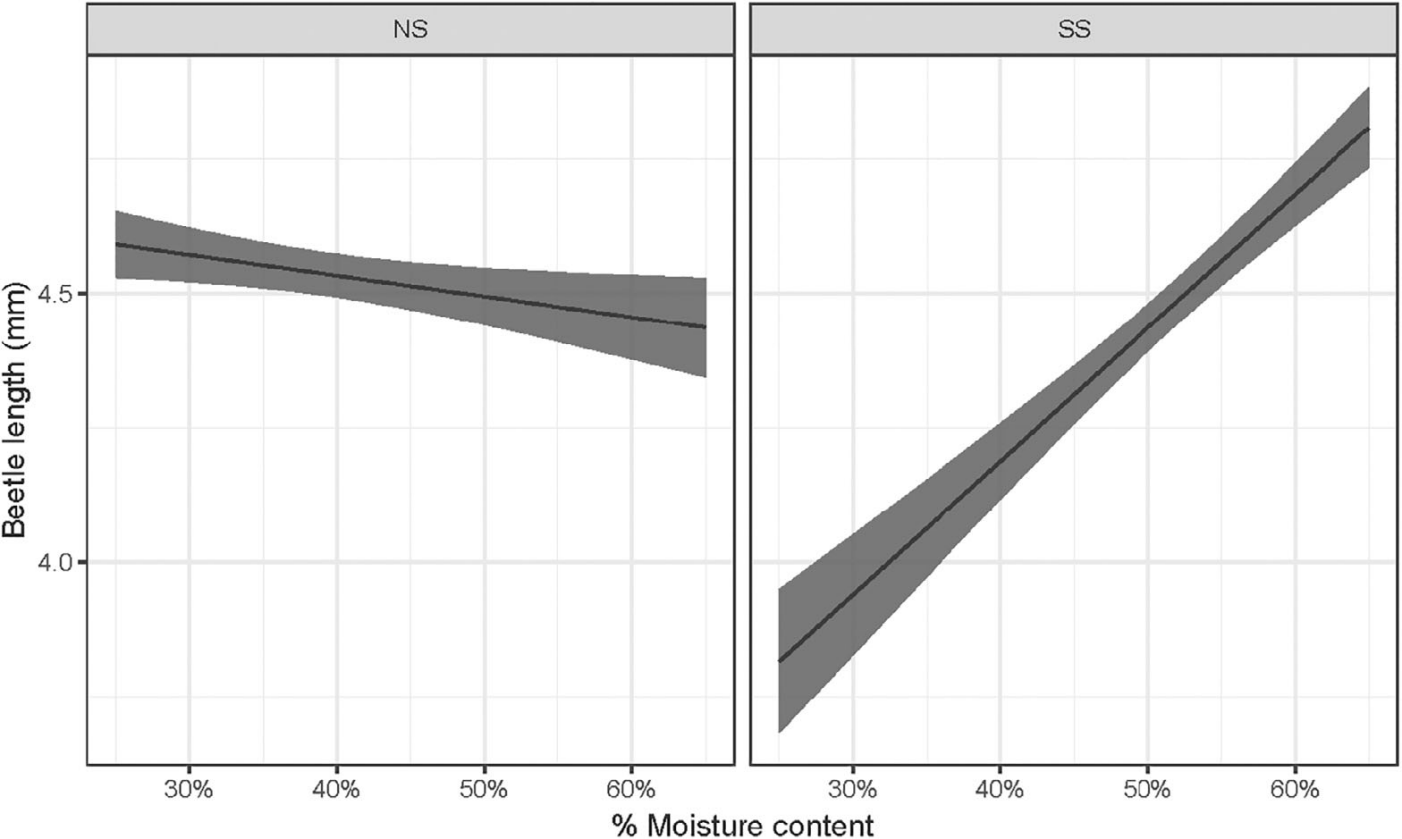
Model‐predicted length of offspring adult beetles developing in the Ardenne field study, according to host species (Norway Spruce (NS) or Sitka Spruce (SS) logs) and initial % moisture content of the bark (mean ± SE).

Overall, SS logs were at least as readily colonized as NS logs at the Ardenne field site (Table 1 and Supporting Information, Table S3). On all of the logs, entrance/emergence holes were present externally and at least one breeding gallery was present under the bark. On average, there were more combined entrance and emergence holes on SS than NS, but this difference was not significant (Table 1, Supporting Information, Table S3). Except on the smallest logs, where breeding galleries were longer on NS than SS, there were more and longer breeding galleries on SS than NS (Table 1 and Supporting Information, Table S3, Fig. S2). Host species did not influence pupal mortality as a main effect, but while numbers of dead pupae remained similar across log sizes in NS, in SS there were more dead pupae on larger logs, and less on smaller logs (Supporting Information, Table S3). On average, beetles that developed on NS had a slightly longer body length, but the largest beetles were found on the SS logs with the highest initial moisture content (Table 1 and Supporting Information, Table S3, Fig. 3).

**Table 1:** Significance of species as a main effect on the response in each of the linear models of the Ardenne field choice experiment data, and raw data mean ± standard error for each response in Norway spruce (NS) and Sitka spruce (SS).

Response	*Χ* ^2^	Df	Pr (>Chisq)	Significance	NS means ± SE (raw data)	SS means ± SE (raw data)
Combined entry and emergence holes	0.26	1	0.61	—	98.83 ± 7.10	116.19 ± 10.84
Length of adult beetles (mm)	7.2	1	0.007	**	4.54 ± 0.02	4.51 ± 0.03
Length of breeding galleries (mm)	42.0	1	<0.0001	***	3308.01 ± 310.17	5542.08 ± 449.96
Number of breeding galleries	6.8	1	0.009	**	54.68 ± 4.86	77.32 ± 5.93
Number of dead pupae	0.74	1	0.39	—	113.36 ± 16.97	102.31 ± 16.81

Scale () indicates that the variables were scaled and centered in the models.

Log positioning (clearfell/forest edge) and layout (open/closed square) affected beetle breeding activity irrespective of species. More and longer breeding galleries and more dead pupae were found in the logs at the forest edge than in the clear‐fell and beetles that developed in the forest edge were larger in body length on average (Table 2). The arrangement of the logs (open or closed square) influenced the number of combined entry and emergence holes, with more holes found on the closed squares where the logs were closer to the pheromone (*n* = 88 ± 8.1, 113 ± 10.2, on open, closed squares, respectively; Supporting Information, Table S3). More dead pupae were found in closed squares (*n* = 61.5 ± 7.6, 86.3 ± 9.4, on open, closed squares, respectively; Supporting Information, Table S3). Distance from the timber stack did not affect any of the response variables (Table S3).

**Table 2:** The influence of forest area type (clear‐fell *vs* forest edge) on *Ips typographus* breeding success and mortality in the Ardenne field study.

Variable	Clear‐fell (response ± SE)	Forest edge (response ± SE)
No. of breeding galleries	43.1 ± 4.8^a^	76 ± 7.8^b^
Length of breeding galleries (mm)	3206 ± 329^a^	5623 ± 328^b^
No. of dead pupae	30.7 ± 5.1^a^	160.2 ± 26.3^b^
Length of new adult beetles (mm)	4.3 ± 0.06^a^	4.5 ± 0.06^b^

Letters denote significant differences (a < b).

**Figure 4:**
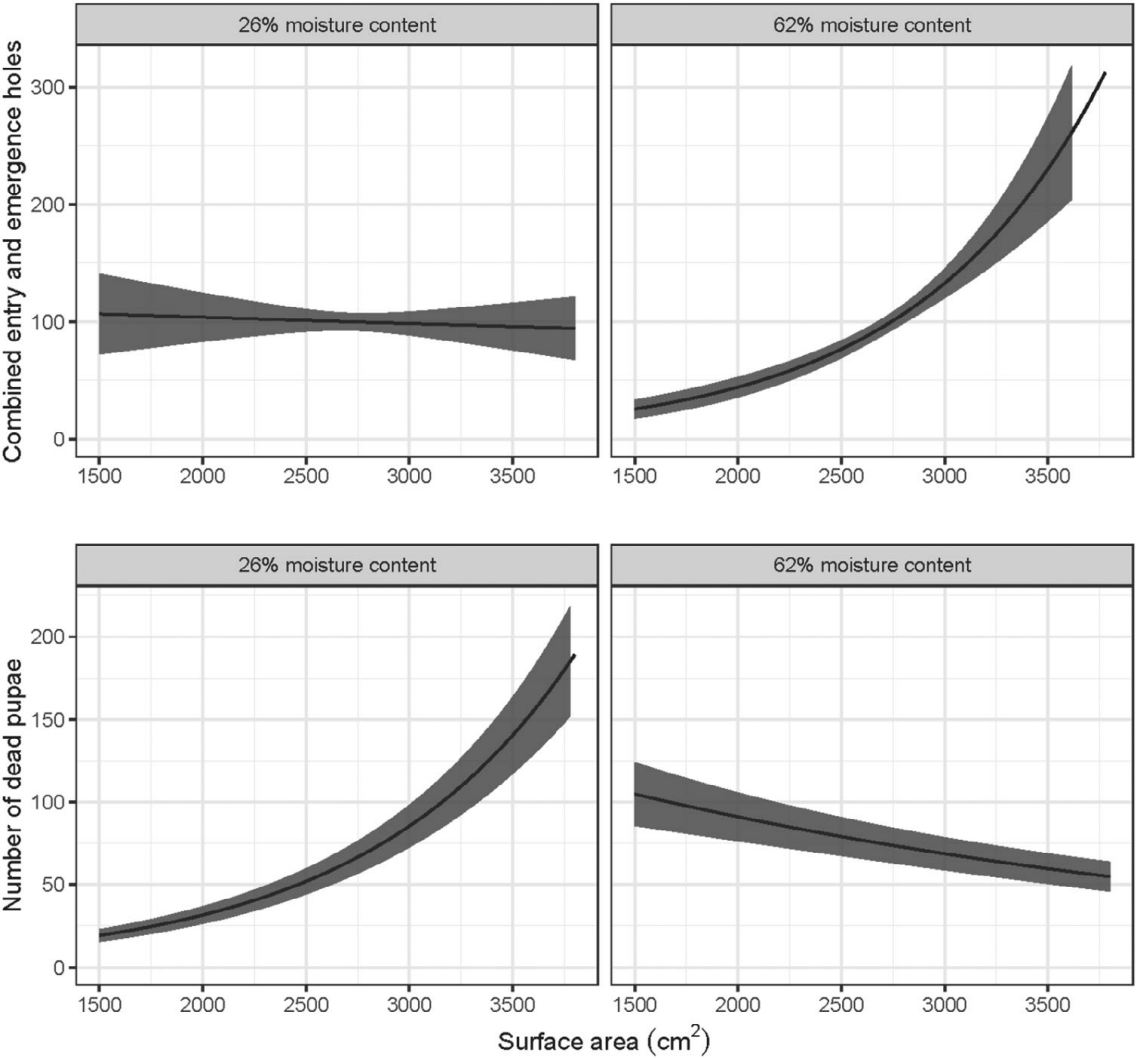
Visualization of the model‐predicted, interacting effects of surface area (cm^2^) and percentage moisture content on the number of combined entry/emergence holes (above) and number of dead pupae (below). Less holes were observed on small billets when initial percentage moisture content was high and mortality of pupae was greater; conversely, more holes were observed on larger billets when percentage initial moisture content was high, because the survival of pupae was greater.

Log surface area and initial % moisture content also influenced colonization and breeding success. Larger logs with a greater surface area had more combined entry and emergence holes, breeding galleries and dead pupae, and longer breeding gallery length on average (Supporting Information, Table S3, Fig. 4). An interaction between log moisture content and surface area exerted an inverse influence on the number of combined entry/emergence holes and the number of dead pupae, respectively: there were less holes on small logs when initial % moisture content was high and mortality of pupae was greater; conversely, there were more holes on larger logs when % initial moisture content was high, because the survival of pupae was greater.

### 3.3 Assessment of beetle behavior in olfactometer experiments

Beetles reared on both NS and SS material showed a similar preference in the olfactometer assays, preferring the odor of spruce billets to blank air (Table 3: Experiment 1–2). Neither beetle group discriminated between aged NS and SS material (Table 3: Experiment 4) but both preferred the odor of freshly cut SS billets over NS billets regardless of which host species they had been reared on (Table 3: Experiment 3). The beetles did not differentiate between the synthetic NS and SS blends in four‐arm olfactometer tests, similarly to behavioral responses given to aged billet material (Table 3: Experiment 5).

**Table 3:** Mean time (min) ± Standard Error (SE) spent by *Ips typographus* individuals in areas of a four‐arm olfactometer flushed with air from different odor sources.

Experiment*	Treatment	NS‐reared beetles			SS‐reared beetles		
		Time mean ± SE	Number of replicates	*F*‐value	Time mean ± SE	Number of replicates	*F*‐value
1	NS billet aged	2.04 ± 0.44	10	0.017	3.38 ± 0.56	10	<0.001
	Control	1.00 ± 0.12			1.29 ± 0.22		
2	SS billet aged	4.95 ± 0.90	10	<0.001	6.40 ± 0.70	10	<0.001
	Control	1.37 ± 0.22			1.57 ± 0.22		
3	NS billet fresh	2.92 ± 0.82b	10	<0.001	3.55 ± 0.49b	10	<0.001
	SS billet fresh	4.67 ± 0.70c			5.56 ± 0.95c		
	Control	1.22 ± 0.23a			1.18 ± 0.22a		
4	NS billet aged	3.34 ± 0.61b	10	<0.001	2.14 ± 0.47ab	10	0.059
	SS billet aged	3.13 ± 0.47b			3.26 ± 0.82b		
	Control	1.56 ± 0.21a			1.40 ± 0.26a		
5	Aged NS synthetic blend	2.00 ± 0.28b	9	0.054	2.77 ± 0.35b	10	0.026
	Aged SS synthetic blend	1.90 ± 0.38ab			2.03 ± 0.36ab		
	Control	1.26 ± 0.17a			1.56 ± 0.23a		

*F*‐values are from the method of residual maximum likelihood (REML) used to fit a linear mixed model for each experiment. Means followed by different letters indicate significant difference for data on the square root scale by least significant difference (LSD) at the 5% (*P* = 0.05) level (Supplementary methods).

* The number of control arms for exp. 1–2 was three, whereas it was two for exp. 3–5. The control was blank air for exp. 1–4, and 10 μL diethyl ether for exp. 5.

### 3.4 Gas chromatography and electrophysiological analysis of NS and SS extracts

Volatile collections from NS and SS spruce billets were performed, and extracts analyzed using GC‐FID to determine quantitative differences across treatments. There was a significant interaction (*P* < 0.019, *F*‐test) between ‘species’ and ‘age’ for headspace concentrations (ng/g fresh weight/h) of sesquiterpene1, sesquiterpene2, sesquiterpene3, longifolene, (*E*)‐caryophyllene, α‐bergamotene, (*E*)‐β‐farnesene, humulene, β‐cubebene and β‐curcumene (Supporting Information, Table S4). There was a significant main effect of ‘species’ (*P* < 0.048, *F*‐test) and ‘age’ (*P* < 0.031, *F*‐test), but no interaction, on headspace concentrations for β‐pinene, myrcene, (*Z*)‐3‐pinanone, α‐longipinene and sesquiterpene5 (Supporting Information, Table S5). There was a main effect of ‘species’ only (*P* < 0.040, *F*‐test) for α‐pinene, camphene and (*E*)‐3‐pinanone, which were significantly greater in NS compared to SS, and sabinene, which was significantly greater in SS (Supporting Information, Table S5). There was a main effect of ‘age’ only (*P* < 0.025, *F*‐test) for β‐thujene, α‐phellandrene, limonene/β‐phellandrene, γ‐terpinene, terpinolene, tridecane and sesquiterpene4, all of which were greater in fresh compared to aged billets (Supporting Information, Table S5). Finally, there were no significant effects (*P* < 0.05, *F*‐test) for 3‐carene, camphor, α‐terpineol, bornyl acetate and α‐curcumene (Supporting Information, Table S5); however, the interaction for α‐terpineol was only marginally non‐significant (*P* = 0.076, *F*‐test).

**Figure 5:**
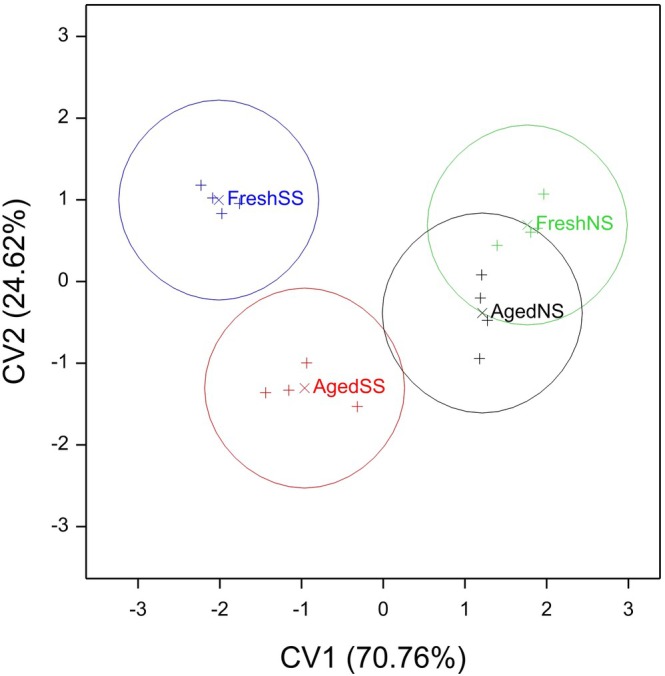
CVA plot showing the separation of the four treatment combinations labelled as Fresh SS, Fresh NS, Aged SS and Aged NS, based on analysis of spruce billet VOC extracts. The CV scores are plotted as ‘+’ and the CV means as ‘×’ with 95% confidence circles shown around the means.

Considering the CVA plot (Fig. 5), CV1 pulls out differences due to ‘species’ and CV2 pulls out differences due to ‘age’. The first two CVs accounted for 95.38% of the variation and hence possible discrimination between the four treatment combinations. CV1 accounted for 70.76% and CV2 for 24.62%. From the CV loadings, for CV1, α‐terpineol and, to a lesser extent, myrcene, (*Z*)‐3‐pinanone and longifolene in the positive direction, and sesquiterpene5 in the negative direction would be seen as most responsible for the species differences. For CV2, limonene/β‐phellandrene in the positive direction and sesquiterpene2 in the negative direction would be seen as most responsible for the age differences. The CVA plot shows that there are greater differences between fresh and aged material for Sitka than for Norway spruce, but with a strong overall separation of the two species (Fig. 5).

**Figure 6:**
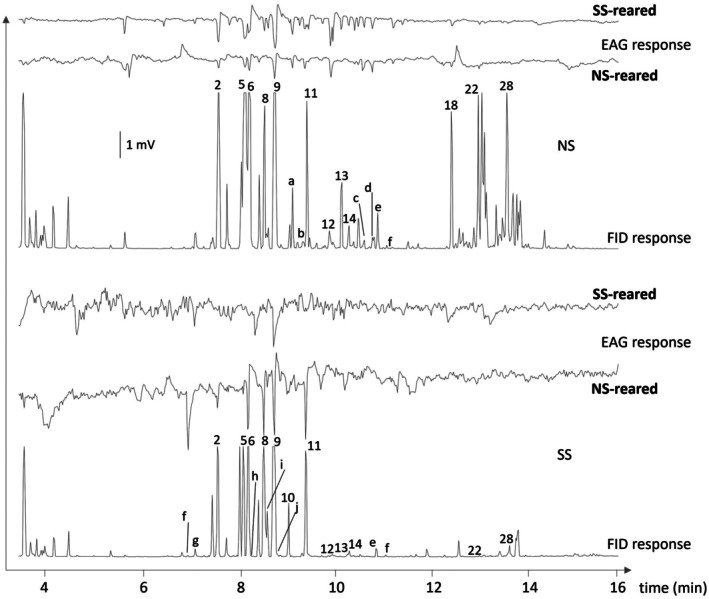
Representative antennal responses of NS‐ and SS‐reared *Ips typographus* to spruce billet headspace extracts in GC‐EAG. Peak numbers refer to compounds in Table S4. 2: α‐pinene, 5: β ‐pinene, 6: myrcene, 7: α‐phellandrene, 8: 3‐carene, 9: limonene/β‐phellandrene, 10: γ‐terpinene, 11: terpinolene, 12: camphor, 13: (*E*)‐pinanone, 14: (*Z*)‐pinanone, 18: α‐longipinene, 22: longifolene, 28: β ‐cubebene, a‐j: unidentified peaks.

NS‐ and SS‐reared beetles showed similar response profiles in GC‐EAG experiments (Fig. 6).

## 4 DISCUSSION

The findings of the laboratory choice, field choice and chemical ecology experiments in this study indicate that adult *I. typographus* find cut Sitka and Norway spruce material similarly attractive, and that cut Sitka spruce is a suitable breeding resource for this pest insect. Whilst European populations of the beetle remain high, additional incursions into southern England *via* aerial dispersal may be expected, and the risk of establishment and spread should be anticipated, including into SS forests.

### 4.1 Colonization of spruce billets

In both laboratory and field experiments, adult *I. typographus* colonized SS and NS billets/logs at a similar frequency. Although some results seem to indicate higher breeding success in one or other host species, according to billet size or moisture content, these appear to balance out across the experiments. Moisture content seemed to be an important and consistent driver of breeding success throughout the study. Our preliminary lab choice experiments demonstrated the influence of initial moisture content on the seasonal suitability of spruce material: the lower bark moisture content of billets cut during winter significantly reduced breeding gallery length and offspring survival as the bark desiccated more rapidly than spring or summer‐cut billets at the same constant temperature (D. Inward & K. Reed unpublished). By autumn, phloem sieve cells in temperate conifers largely cease to function^40^ as growth ends and cold acclimation begins, leading to a reduction in bark moisture content. Subsequent assays were therefore conducted during the spring and summer according to availability of newly emerged beetles.

Under the field conditions investigated in the Ardenne, it is clear that freshly cut SS offers a suitable and effective breeding resource for *I. typographus*. All NS and SS logs were colonized and the numbers of entry and emergence holes were similar across host species. Whereas total numbers and lengths of breeding galleries per log were higher on SS than NS, offspring collected from the NS billets were typically larger than from the SS logs, although greater intraspecific competition might have reduced offspring size in the SS. The initial bark moisture content appeared to influence breeding success and offspring survival in the field trial, interacting with species and log size. Bark moisture more strongly influenced offspring size in SS than NS, which may indicate a more finite window of moisture suitability in cut material of this species. On both NS and SS, more entry and emergence holes were observed on billets with greater initial moisture content, as well as on larger billets, which presumably retained their moisture for longer and perhaps offered more protection from the heatwave conditions present during the study. Higher bark moisture levels can influence fungal and microbial activity, however,^41^ which may increase offspring mortality. Colonization of all logs was less frequent in the clearfell area compared to the shaded forest edge, likely due to the high and prolonged summer temperatures experienced during July 2022 across much of Europe,^42^ which greatly exceeded the optimal breeding temperature (~ 29 °C) of *I. typographus*.^43^ These heatwave temperatures likely contributed to the pupal mortality observed in many of the logs of both spruce species. Our findings contrast somewhat with the reduced breeding recorded on SS in the no‐choice experiment of Flø *et al*.^11^; the free‐choice design of our study may have enhanced host acceptance of SS. There is also likely to be geographical and provenance variation in the quality of SS as a host for *I. typographus*, as we observed in our choice experiments. Our results indicate that SS logs may be used for breeding by *I. typographus*, and this has important implications for felled SS timber which is stored in the forest, and for wind‐snapped or windthrown trees. However, assumptions should not be made regarding the susceptibility of live SS trees, which will exhibit induced defenses not present in cut material. The few field observations of colonized SS trees to date indicate only that they were present in close association with attacked NS trees, and that the SS trees were either recently felled, predisposed by butt‐rot, or subject to a large outbreaking beetle population before being colonized.

### 4.2 Host selection

Our behavioral tests indicate that the first phase of host selection, host search, in *I. typographus* is at least in part mediated by tree volatile organic compounds (VOCs). The behavioral preference for fresh SS over fresh NS might be explained by the different headspace composition of the two spruce species. In aged wood, such differences may not be large enough for the beetles to differentiate between species. CVA analyses highlighted several VOCs that contribute to the observed differences in host chemical profiles and which are EAG‐active, indicating they may play a key role in *I. typographus* host searching behavior. Kandasamy *et al*.^44^ showed that *I. typographus* possesses dedicated olfactory sensory neurons for oxygenated metabolites, such as camphor, isopinocamphone, pinocamphone and (±)‐pinocarvone, which attract beetles.

These findings corroborate our results on the EAG‐active oxygenated monoterpenes camphor and (*E/Z*)‐pinanone, of which camphor was a minor constituent of the synthetic blend found to be preferred by the beetles in behavioral assays. It should be noted that the implications of our results are correlative between field colonization of logs and behavioral experiments on VOCs, and field trials with the synthetic blends would be required to determine that these VOCs indeed attract the beetles to trees in the natural environment.

Although the role of spruce VOCs in host selection by *I. typographus* is poorly understood,^14^ they are likely to be of key importance for host‐colonizing males (primary attraction), as they are in other Scolytinae species.^18^, ^45^ The host monoterpene (−)‐α‐pinene synergizes the attractiveness of the *I. typographus* aggregation pheromone,^46^ and the species possesses olfactory receptors tuned to host monoterpenes.^17^, ^47^ The dispersal of *I. typographus* is influenced by the presence of freshly cut spruce material,^48^ which Franklin *et al*.^27^ suggest is attractive to beetles up to a range of approximately 250 m. Hedgren *et al*.^49^ attribute this to greater monoterpene production from freshly felled than from 1‐month‐old spruce material. We found that fresh NS and SS billets released monoterpenoids approximately 2.5 and 3.5 times more than aged billets, respectively. Interestingly, a field trapping study by Lindelöw *et al*.^50^ found that *I. typographus* was strongly attracted to aged, but only weakly to freshly felled, NS wood material, perhaps reflecting an increased suitability of the resource for breeding as it ages. Although we did not behaviorally compare VOC extracts from fresh and aged billets within either species directly, the results by Lindelöw *et al*.^50^ indicate that the olfactory behavior elicited by aged spruce billets may be important to evaluate preference traits in host selection. Most of the VOCs identified here have been reported previously from NS bark surface extracts,^17^ and α‐ and β‐pinene, myrcene, limonene, β‐phellandrene, camphor and piperitone have been identified from *P. sitchensis* bark.^51^, ^52^ It should be noted, however, that aggregation pheromone communication channels^15^ can also interfere with beetle perception of tree VOCs, with several studies demonstrating green leaf volatiles from non‐host trees reduce attraction of *I. typographus* to their aggregation pheromone.^12^, ^53^

Finally, the similar EAG and behavioral responses of NS‐ and SS‐reared beetles suggest induced host preference does not seem to play a major role in host selection by *I. typographus*. Induced food preference^23^, ^54^ occurs when an insect's preference increases for the plant species they were reared upon, an effect which may be maintained throughout a number of instars.

## 5 CONCLUSION

The results of this study, which combines experimental choice assays with a thorough examination of the chemical ecology underpinning the host search mechanism, suggest *I. typographus* finds fresh‐cut SS material as attractive and suitable for breeding as NS. The findings highlight the role of VOCs in host location by *I. typographus*, and we may assume that the beetle may locate and colonize felled (or wind‐snapped) SS as readily as cut NS, which may increase its establishment risk in SS‐growing regions. These results are concerning when considered in combination with the recent aerial dispersal of *I. typographus* into England and subsequent establishments in NS forest. Forest management practices employed to limit *I. typographus* population build‐up in Norway spruce forests are likely to become similarly necessary for Sitka spruce growers, in particular the prompt removal of felled timber or wind‐damaged trees from the forest. There are also risk implications for Sitka spruce worldwide; recent modelling suggests that the climate within much of the native range of Sitka spruce is highly suitable for establishment by the beetle,^55^ although it has not established in North America despite frequent port interceptions.^56^ It is important, however, to recognize that the interactions reported in this study refer to cut tree material only, and that reports to date of *I. typographus* colonizing SS refer to cut logs and felled trees,^4^, ^11^, ^57^ and not live SS trees. Cut logs will have significantly reduced defensive capabilities compared to live trees, with induced (secondary) defenses becoming rapidly non‐functional. For example, *Picea* have relatively few constitutive (pre‐existing) resin ducts compared to *Pinus* species,^58^ and the rapid formation of induced resin ducts appears to be a key defense against bark beetle attack.^59^ Such responses will be severely disabled in cut material. Any assessment of the susceptibility of SS to *I. typographus*, or the potential for population growth and outbreak in SS forest, therefore, remains incomplete without additional understanding of the interactions of the beetle with live SS trees.

## CONFLICT OF INTEREST

The authors have no conflicts of interest to declare.

## Supporting information


**Data S1.** Supporting Information.

## Data Availability

The data that support the findings of this study are available from the corresponding author upon reasonable request. Data collected during this study, including the volatile organic compounds collected by air entrainment from Norway and Sitka spruce material, and the behavioural response to these compounds of *Ips typographus* within the four‐arm olfactometer, is available from the Rothamsted Research data repository: https://doi.org/10.23637/mhvmi6az
