# Supplementary material for: Investigating the threat to Sitka spruce from Ips typographus : discrimination and colonization of Britain's principal commercial conifer by a damaging forest pest
Source: Pest Manag Sci. 2025 Jan 27;81(11):7448–60. doi: 10.1002/ps.8644 (PMC12539388; doi:10.1002/ps.8644)
Supplement: Supplementary file 1 — Data S1. Supporting Information. [file PS-81-7448-s001.docx]

**Supplementary Material: ‘*Investigating the threat to Sitka spruce from Ips typographus: discrimination and colonisation of Britain’s principal commercial conifer by a damaging forest pest’.***

Daegan Inward^1^, Jozsef Vuts^2^, Gareth Thomas^2^, Kerry Barnard^1^, John C. Caulfield^2^, Stephen J. Powers^3^, Ana Uglow^1^, Katy Reed^1^

^1^ Forest Research, Alice Holt Lodge, Farnham, Surrey, GU10 4LH, U.K.

^2^ Protecting Crops and the Environment, Rothamsted Research, Harpenden, AL5 2JQ, UK

^3^ Stats Powers Ltd, Somerset, UK,

Figure S1. Mean time (min) ± Standard Error (SE) spent by *I. typographus* individuals in areas of a four-arm olfactometer flushed with air from different odour sources. Colours indicate the proportion of females and males of the total number of beetles tested.


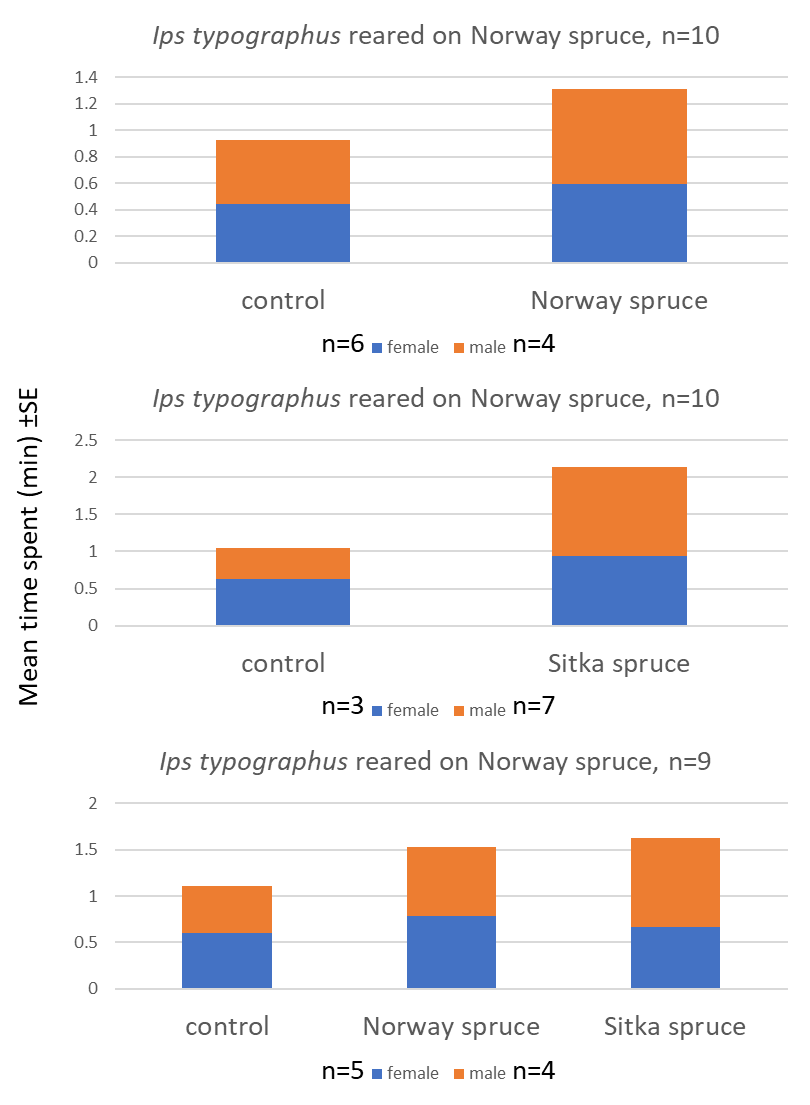


Figure S2. Total (cumulative) breeding gallery length per log (model-predicted) in the Ardenne field experiment, according to host species (Norway Spruce (NS) or Sitka Spruce (SS)) and log surface area (mean ± SE).


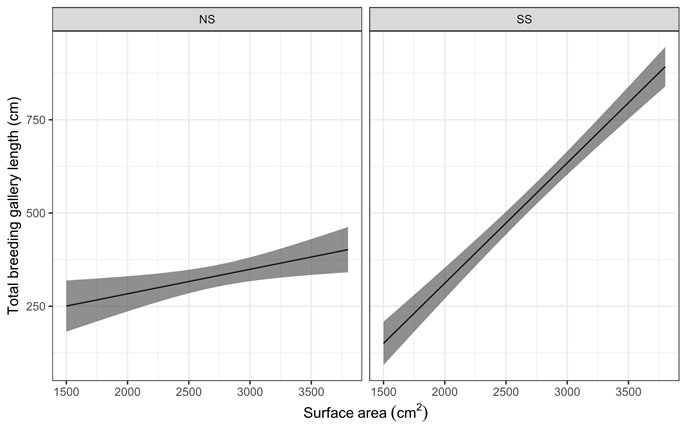


Table S1. Differences in the proportions of galleries in Norway spruce to Sitka spruce billets in the laboratory choice experiment; total galleries (breeding + non-breeding), breeding galleries, and non-breeding galleries.

| **Response** | **Df** | **X^2^** | ***p*** |
| --- | --- | --- | --- |
| Total number of galleries | 1 | 0.0251 | 0.87 |
| Breeding galleries | 1 | 0.46 | 0.5 |
| Non-breeding galleries | 1 | 0.67 | 0.42 |

Table S2. Influence of the explanatory variables in the laboratory choice experiment; host species (Norway Spruce vs Sitka Spruce), billet diameter, and their interactions, on total breeding gallery length per billet and the length of individual breeding galleries.

| **Response** | **explanatory variable** | **Df** | **X^2^** | ***p*** |
| --- | --- | --- | --- | --- |
| Total breeding gallery length per billet | Spruce species | 1 | 2.14 | 0.14 |
|  | scale (diameter) | 1 | 0.44 | 0.51 |
|  | Spruce species : scale (diameter) | 1 | 0.49 | 0.49 |
| Individual breeding gallery length | Spruce species | 1 | 5.11 | 0.02 |
|  | scale (diameter) | 1 | 0.66 | 0.42 |
|  | Spruce species : scale (diameter) | 1 | 1.67 | 0.2 |

Table S3. Influence of the explanatory variables in the Field choice experiment: host species (Norway Spruce vs Sitka Spruce), % bark moisture content, billet surface area, and their interactions, plot type (clearfell or forest edge), distance from the billets to the pheromone lure (open or closed square), and distance from the source log pile (m), on the responses: combined entry and emergence holes, number of breeding galleries per billet, length of breeding galleries per billet, number of dead pupae per billet, and length of newly-developed adults. Scale () indicates that the variables were scaled and centred in the models.

| **combined entry and emergence holes** | **Explanatory variable** | **Χ^2^** | **Df** | **Pr(>Chisq)** | **Significance** |
| --- | --- | --- | --- | --- | --- |
| combined entry and emergence holes | species | 0.26 | 1 | 0.61 |  |
|  | scale(% bark moisture content) | 1.04 | 1 | 0.31 |  |
|  | scale(surface area (cm2)) | 23.8 | 1 | < 0.0001 | *** |
|  | open or closed square | 5.8 | 1 | 0.016 | * |
|  | area type (clearfell or forest edge) | 0.12 | 1 | 0.73 |  |
|  | scale(distance (m)) | 1.38 | 1 | 0.24 |  |
|  | scale(surface area (cm2)):scale(% bark moisture content) | 6.9 | 1 | 0.009 | ** |
|  | species:scale(surface area (cm2)) | 0.79 | 1 | 0.37 |  |
|  | species:scale(% bark moisture content) | 0.77 | 1 | 0.38 |  |
|  | species:scale(% bark moisture content):scale(surface area (cm2)) | 0.74 | 1 | 0.39 |  |
| Length of adult beetles (mm) | species | 7.2 | 1 | 0.007 | ** |
|  | scale(% bark moisture content) | 4.2 | 1 | 0.041 | * |
|  | scale(distance (m)) | 0.11 | 1 | 0.74 |  |
|  | open or closed square | 0.01 | 1 | 0.91 |  |
|  | area type (clearfell or forest edge) | 10.9 | 1 | < 0.0001 | *** |
|  | scale(% bark moisture content):scale(surface area (cm2)) | 0.13 | 1 | 0.72 |  |
|  | scale(surface area (cm2)) | 0.33 | 1 | 0.57 |  |
|  | species:scale(surface area (cm2)) | 0.03 | 1 | 0.86 |  |
|  | species:scale(% bark moisture content) | 27.1 | 1 | < 0.0001 | *** |
|  | species:scale(% bark moisture content):scale(surface area (cm2)) | 3.23 | 1 | 0.07 |  |
| length of breeding galleries (mm) | species | 42.0 | 1 | < 0.0001 | *** |
|  | scale(% bark moisture content) | 2.54 | 1 | 0.11 |  |
|  | scale(distance (m)) | 0.01 | 1 | 0.93 |  |
|  | open or closed square | 2.40 | 1 | 0.12 |  |
|  | area type (clearfell or forest edge) | 29.8 | 1 | < 0.0001 | *** |
|  | scale(% bark moisture content):scale(surface area) | 0.01 | 1 | 0.93 |  |
|  | scale(surface area (cm2)) | 53.3 | 1 | < 0.0001 | *** |
|  | species:scale(surface area (cm2)) | 17.7 | 1 | < 0.0001 | *** |
|  | species:scale(% bark moisture content) | 0.00 | 1 | 0.98 |  |
|  | species:scale(% bark moisture content):scale(surface area (cm2)) | 0.35 | 1 | 0.55 |  |
| number of breeding galleries | species | 6.8 | 1 | 0.009 | ** |
|  | scale(% bark moisture content) | 0.08 | 1 | 0.77 |  |
|  | scale(distance (m)) | 0.12 | 1 | 0.73 |  |
|  | open or closed square | 2.50 | 1 | 0.11 |  |
|  | area type (clearfell or forest edge) | 15.8 | 1 | < 0.0001 | *** |
|  | scale(% bark moisture content):scale(surface area) | 0.13 | 1 | 0.71 |  |
|  | scale(surface area (cm2)) | 23.1 | 1 | < 0.0001 | *** |
|  | species:scale(surface area) | 0.96 | 1 | 0.33 |  |
|  | species:scale(% bark moisture content) | 0.36 | 1 | 0.55 |  |
|  | species:scale(% bark moisture content):scale(surface area (cm2)) | 0.21 | 1 | 0.65 |  |
| number of dead pupae | species | 0.74 | 1 | 0.39 |  |
|  | scale(% bark moisture content) | 0.11 | 1 | 0.74 |  |
|  | scale(distance (m)) | 0.05 | 1 | 0.82 |  |
|  | open or closed square | 0.79 | 1 | 0.37 |  |
|  | area type (clearfell or forest edge) | 73.2 | 1 | < 0.0001 | *** |
|  | scale(% bark moisture content):scale(surface area (cm2)) | 51.5 | 1 | < 0.0001 | *** |
|  | scale(surface area (cm2)) | 90.2 | 1 | < 0.0001 | *** |
|  | species:scale(surface area (cm2)) | 32.2 | 1 | < 0.0001 | *** |
|  | species:scale(% bark moisture content) | 0.10 | 1 | 0.76 |  |
|  | species:scale(% bark moisture content):scale(surface area (cm2)) | 1.27 | 1 | 0.26 |  |

Table S4. Mean ±SE ng compound/g fresh weight/h values of volatiles identified from aged and freshly cut NS and SS billets. *Kováts index. ^a^co-eluting.

| Number | compound | KI* | NS fresh | | NS aged | | SS fresh | | SS aged | |
| --- | --- | --- | --- | --- | --- | --- | --- | --- | --- | --- |
|  |  |  | mean | SE | mean | SE | mean | SE | mean | SE |
| 1 | β-thujene | 927 | 0.24 | 0.05 | 0.17 | 0.05 | 0.75 | 0.26 | 0.52 | 0.17 |
| 2 | α-pinene | 936 | 54.82 | 13.97 | 38.55 | 3.50 | 12.63 | 1.31 | 9.60 | 1.41 |
| 3 | camphene | 948 | 2.37 | 1.69 | 1.19 | 0.45 | 0.20 | 0.06 | 0.13 | 0.03 |
| 4 | sabinene | 969 | 0.44 | 0.13 | 0.55 | 0.27 | 6.37 | 3.21 | 3.07 | 1.21 |
| 5 | β-pinene | 978 | 122.60 | 33.77 | 73.08 | 12.53 | 16.44 | 1.61 | 9.69 | 1.18 |
| 6 | myrcene | 987 | 84.40 | 24.65 | 21.19 | 3.72 | 39.83 | 5.01 | 7.57 | 2.24 |
| 7 | α-phellandrene | 999 | 0.98 | 0.07 | 0.36 | 0.06 | 1.01 | 0.09 | 0.23 | 0.06 |
| 8 | 3-carene | 1007 | 24.75 | 11.66 | 14.71 | 6.20 | 10.82 | 10.32 | 3.19 | 2.83 |
| 9 | limonene/β-phellandrene^a^ | 1029 | 161.52 | 42.07 | 41.20 | 19.06 | 150.22 | 7.38 | 31.84 | 8.89 |
| 10 | γ-terpinene | 1053 | 1.07 | 0.25 | 0.39 | 0.27 | 1.04 | 0.33 | 0.22 | 0.09 |
| 11 | terpinolene | 1082 | 7.70 | 1.98 | 2.95 | 1.97 | 13.01 | 3.52 | 2.40 | 0.93 |
| 12 | camphor | 1126 | 0.24 | 0.08 | 0.18 | 0.08 | 0.21 | 0.05 | 0.09 | 0.01 |
| 13 | (E)-3-pinanone | 1143 | 1.20 | 0.44 | 0.41 | 0.05 | 0.13 | 0.05 | 0.11 | 0.03 |
| 14 | (Z)-3-pinanone | 1156 | 0.63 | 0.12 | 0.35 | 0.11 | 0.26 | 0.04 | 0.22 | 0.02 |
| 15 | α-terpineol | 1178 | 0.41 | 0.15 | 0.11 | 0.03 | 0.12 | 0.05 | 0.21 | 0.05 |
| 16 | bornyl acetate | 1275 | 0.24 | 0.08 | 0.46 | 0.38 | 0.11 | 0.04 | 0.07 | 0.01 |
| 17 | tridecane | 1299 | 0.09 | 0.02 | 0.04 | 0.01 | 0.12 | 0.03 | 0.09 | 0.02 |
| 18 | α-longipinene | 1363 | 2.24 | 1.87 | 0.45 | 0.25 | 0.08 | 0.02 | 0.06 | 0.01 |
| 19 | sesquiterpene1 | 1380 | 0.44 | 0.21 | 0.11 | 0.03 | 0.02 | 0.01 | 0.11 | 0.02 |
| 20 | sesquiterpene2 | 1384 | 0.41 | 0.16 | 0.24 | 0.11 | 0.02 | 0.00 | 0.13b | 0.01 |
| 21 | sesquiterpene3 | 1409 | 0.59 | 0.16 | 0.26 | 0.08 | 0.03 | 0.01 | 0.15 | 0.02 |
| 22 | longifolene | 1419 | 3.54 | 1.09 | 1.27 | 0.66 | 0.06 | 0.03 | 0.21 | 0.02 |
| 23 | (E)-caryophyllene | 1430 | 3.38 | 1.94 | 0.74 | 0.21 | 0.09 | 0.01 | 0.16 | 0.03 |
| 24 | α-bergamotene | 1441 | 1.26 | 0.72 | 0.32 | 0.12 | 0.01 | 0.00 | 0.06 | 0.01 |
| 25 | (E)-β-farnesene | 1451 | 0.75 | 0.40 | 0.29 | 0.09 | 0.17 | 0.04 | 0.30 | 0.03 |
| 26 | humulene | 1465 | 0.45 | 0.21 | 0.17 | 0.04 | 0.02 | 0.00 | 0.14 | 0.02 |
| 27 | α-curcumene | 1467 | 0.31 | 0.12 | 0.19 | 0.09 | 0.08 | 0.02 | 0.05 | 0.01 |
| 28 | β-cubebene | 1489 | 5.26 | 4.32 | 0.66 | 0.39 | 0.22 | 0.05 | 0.13 | 0.02 |
| 29 | β-curcumene | 1507 | 0.69 | 0.35 | 0.19 | 0.07 | 0.03 | 0.01 | 0.05 | 0.02 |
| 30 | sesquiterpene4 | 1515 | 0.93 | 0.52 | 0.22 | 0.08 | 1.24 | 0.32 | 0.62 | 0.12 |
| 31 | sesquiterpene5 | 1524 | 0.80 | 0.33 | 0.20 | 0.06 | 1.78 | 0.45 | 0.86 | 0.17 |
|  | TOTAL |  | 532.91 | | 219.82 | | 322.03 | | 91.34 | |

Table S5. F-test p-values from ANOVA for the main effect of `species`, the main effect of `age` and the interaction between these two factors for the 31 chemicals.

| **Chemical** | **p-values** | | |
| --- | --- | --- | --- |
|  | **Species** | **Age** | **Species × Age** |
| β-thujene | 0.195 | 0.025 | 0.861 |
| α-pinene | <0.001 | 0.184 | 0.963 |
| camphene | 0.009 | 0.251 | 0.798 |
| sabinene | 0.040 | 0.345 | 0.258 |
| β-pinene | <0.001 | 0.031 | 0.888 |
| myrcene | 0.010 | <0.001 | 0.317 |
| α-phellandrene | 0.147 | <0.001 | 0.217 |
| 3-carene | 0.073 | 0.224 | 0.870 |
| limonene/β-phellandrene | 0.916 | <0.001 | 0.762 |
| γ-terpinene | 0.691 | 0.001 | 0.940 |
| terpinolene | 0.656 | 0.006 | 0.601 |
| camphor | 0.349 | 0.390 | 0.558 |
| (*E*)-3-pinanone | 0.002 | 0.117 | 0.244 |
| (*Z*)-3-pinanone | 0.048 | 0.026 | 0.121 |
| α-terpineol | 0.535 | 0.672 | 0.076 |
| bornyl acetate | 0.274 | 0.454 | 0.851 |
| tridecane | 0.107 | 0.019 | 0.160 |
| α-longipinene | 0.042 | 0.020 | 0.089 |
| sesquiterpene1 | 0.009 | 0.186 | <0.001 |
| sesquiterpene2 | 0.029 | 0.040 | 0.003 |
| sesquiterpene3 | 0.003 | 0.098 | <0.001 |
| longifolene | 0.001 | 0.677 | 0.003 |
| (*E*)-caryophyllene | 0.005 | 0.181 | 0.004 |
| α-bergamotene | 0.001 | 0.190 | <0.001 |
| (*E*)-β-farnesene | 0.277 | 0.614 | 0.002 |
| humulene | 0.007 | 0.023 | <0.001 |
| α-curcumene | 0.102 | 0.135 | 0.694 |
| β-cubebene | 0.106 | 0.001 | 0.022 |
| β-curcumene | 0.008 | 0.198 | 0.019 |
| sesquiterpene4 | 0.174 | 0.019 | 0.475 |
| sesquiterpene5 | 0.029 | 0.002 | 0.158 |

**Supplementary methods**

Statistical analysis of the volatile organic compound (VOC) composition of spruce logs (also see sections 2.6 and 3.4). Analysis of Variance (ANOVA) was used to determine the statistical significance of the effect of species, age, and the interaction between these two factors on VOCs emitted by spruce logs. The analysis took account of the two samples from each tree, using a samples-from-main-plots design for the experiment, thus testing the main effect of species at the level of individual trees, and the main effect of aging and the species by aging interaction at the level of samples within trees. Relevant tables of means were output for comparison of pairs of means of biological interest, using the standard error of the difference (SED) between them on the appropriate residual degrees of freedom from the ANOVA, by way of the least significant difference (LSD) at the 5% (p = 0.05) level of significance. Inspection of residual plots revealed that natural log transformation of the ng compound/h/g spruce billet fresh weight data was required, the assumptions of the analysis being satisfied with the data on this scale.

Canonical variates analysis (CVA) (also see sections 2.6 and 3.4) was used to analyse all the spruce log VOC data together (on the log-scale, as required from the ANOVA) and discriminate between the four treatment combinations (two species by two ages). The method finds linear combinations (i.e. canonical variates, CVs) of the chemicals that maximise the ratio of the between treatment combinations variation to the within treatment combinations variation, thus performing a discrimination between all treatment combinations. The fewest number of CVs are retained that take up the most variation in the data and hence make the most discrimination. The data are then visualised on the new dimensions, by plotting the CV scores for each sample. The mean of CV scores in each dimension, for each treatment combination (i.e. the CV means) are also plotted. Making the assumption of a multivariate Normal distribution for the data, 95% confidence circles are placed around the CV means for the treatment combinations. It is noted that the radius of these circles is $\sqrt{\chi_{2,0.05}^{2}}/\sqrt{n}$, where *n* = 4 (the replication) and $\chi_{2,0.05}^{2}$ = 5.99 is the upper 5% point of a Chi-squared distribution on 2 degrees of freedom. Non-overlapping confidence circles give evidence of significant differences between treatment combinations at the p = 0.05 level of significance. The magnitude of CV loadings on the chemicals can be inspected to see which ones are important in the discrimination observed in the particular CV directions. All analyses were performed using the Genstat statistics package (22^nd^ edition, © VSN International Ltd, Hemel Hempstead, UK).
